# Association Between Vitamin D, Vitamin B12 and Folate Levels and Persistent Subclinical Inflammation in Pediatric Familial Mediterranean Fever

**DOI:** 10.3390/children13030371

**Published:** 2026-03-05

**Authors:** Büşra Tetik Dinçer, Esma Akboğa, Fazilet Melikoğlu, Gül Özçelik

**Affiliations:** 1 Department of Pediatrics, Ankara Etlik City Hospital, 06170 Ankara, Türkiye; 2 Department of Pediatrics, University of Health Sciences, Şişli Hamidiye Etfal Training and Research Hospital, 34371 İstanbul, Türkiye; 3 Department of Pediatrics, Division of Pediatric Nephrology, University of Health Sciences, Şişli Hamidiye Etfal Training and Research Hospital, 34371 İstanbul, Türkiye

**Keywords:** familial Mediterranean fever, micronutrients, persistent subclinical inflammation, vitamin D

## Abstract

**Background/Objectives**: Familial Mediterranean fever (FMF) is the most common periodic fever syndrome worldwide, and persistent subclinical inflammation has been reported in 10–20% of cases during attack-free periods. Although the immunomodulatory effects of vitamin D, vitamin B12 and folate have been investigated in various conditions, data on their relationship with subclinical inflammation in FMF patients remain limited. This study aimed to evaluate the association between micronutrient levels and subclinical inflammation in pediatric FMF. **Methods**: Children aged 2–18 years with an FMF diagnosis of more than two years, receiving regular colchicine therapy, and attack-free for at least two months were included. Patients with other autoinflammatory diseases, colchicine resistance, concomitant renal disease, active infection, or inadequate follow-up were excluded. Demographic, clinical, genetic, and biochemical data were analyzed. **Results**: A total of 253 patients were included, with a median age of 14 years (range, 3–18), and 133 (52.6%) were female. Persistent subclinical inflammation was observed in 31 patients (12.3%). Genetic analysis revealed homozygous M694V mutations in 71 patients (28%). Median vitamin levels were as follows: vitamin D 17.3 ng/mL (IQR 10.6–27.1), vitamin B12 288 pg/mL (IQR 214–367), and folate 6.4 ng/mL (IQR 4.8–7.6). Comparison between patients with and without subclinical inflammation showed no significant differences in micronutrient levels. **Conclusions**: Although micronutrients have been reported to play immunomodulatory roles, we did not observe significant association between vitamin levels and subclinical inflammation in pediatric FMF patients in our study.

## 1. Introduction

Familial Mediterranean fever (FMF) is the most common hereditary periodic fever syndrome, characterized by recurrent episodes of fever and serositis [1]. It predominantly affects populations of Turkish, Arab, Armenian, and Jewish descent. Attacks usually last 1–3 days, and patients are typically asymptomatic between episodes. However, persistent subclinical inflammation has been reported in approximately 10–20% of patients during attack-free periods [2,3]. In some patients, persistent subclinical inflammation occurs as the only manifestation, which increases the risk of severe complications such as amyloidosis, a potentially fatal condition [4]. Several studies have investigated factors associated with persistent subclinical inflammation, yet the results remain inconsistent [5,6,7]. It has also been suggested that persistent subclinical inflammation is related to colchicine resistance, and identifying risk factors associated with this condition could be useful for optimizing treatment strategies [8,9]. Although the immunomodulatory effects of micronutrients have been widely studied, data regarding the relationship between micronutrient levels and subclinical inflammation in FMF patients are lacking [10,11,12]. The aim of this study was to investigate the association between persistent subclinical inflammation and serum levels of vitamin D, vitamin B12, and folate in pediatric patients with FMF.

## 2. Materials and Methods

This study included patients with FMF who were admitted to our center, had been on regular colchicine treatment and had not experienced an attack for more than two months. The study was conducted in accordance with the principles of the Declaration of Helsinki and approved by the Clinical Research Ethics Committee of Şişli Etfal Training and Research Hospital (17 June 2025; Decision No: 3053).

Patients aged 2–18 years with a diagnosis of FMF for more than two years, on regular colchicine therapy, and attack-free for at least two months were included. Exclusion criteria were the presence of autoinflammatory diseases other than FMF, a previous FMF attack in the last two months, comorbid conditions involving the renal system other than FMF, active infection, and noncompliance with follow-up. Blood test results of study participants obtained between January 2023 and December 2023 were retrospectively reviewed. Demographic and laboratory data including age, sex, hemoglobin level, mean corpuscular volume (MCV), neutrophil count, lymphocyte count, neutrophil-to-lymphocyte ratio (NLR), erythrocyte sedimentation rate (ESR), ferritin level, presence of iron deficiency anemia, C-reactive protein (CRP), serum amyloid A (SAA) protein, vitamin D, vitamin B12 and folate levels were evaluated.

The diagnosis of FMF was established based on the Tel-Hashomer criteria. Colchicine dosing in our center follows the recommendations of the European Alliance of Associations for Rheumatology (EULAR), with age-adjusted daily doses defined as ≤0.5 mg/day for children aged 5–10 years and 1.0–1.5 mg/day for those older than 10 years. Serum 25-hydroxyvitamin D levels were measured using a chemiluminescent immunoassay method (Beckman Coulter, Brea, CA, USA). Serum vitamin B12 and folate levels were determined by electrochemiluminescence immunoassay using commercially available kits (Beckman Coulter, CA, USA). CRP levels were measured using an immunoturbidimetric method (Beckman Coulter, CA, USA), and SAA concentrations were assessed by nephelometric assay using standardized commercial kits (Siemens Healthineers, Erlangen, Germany). In our center, routine genetic testing for FMF includes sequencing of exons 2, 3, 5, and 10 of the Mediterranean fever (MEFV) gene, which cover the majority of pathogenic variants associated with the disease. Patients who did not carry any of the screened MEFV variants in exons 2, 3, 5, and 10 were classified as wild-type. FMF diagnosis was based on the Tel-Hashomer criteria [13]. Persistent subclinical inflammation was defined as elevated CRP, ESR and/or SAA levels in ≥75% of measurements obtained during attack-free periods, in the absence of any identifiable cause [14]. The upper limits of normal in our laboratory were CRP: 5 mg/dL, SAA: 10 mg/L, ESR: 20 mm/h, and NLR: 1.65. The diagnosis of iron deficiency anemia was established based on the hemoglobin thresholds defined by the World Health Organization [15].

### Statistical Analysis

All analyses were performed using SPSS (Statistical Package for the Social Sciences), version 25.0 (IBM Corp., Armonk, NY, USA). Descriptive statistics (numbers, percentages, means, medians, and interquartile ranges [IQRs]) were used to summarize the data. Categorical variables were compared using the Chi-square test, while continuous variables were compared using the Mann–Whitney U-test. A *p*-value of <0.05 was considered statistically significant at a 95% confidence interval.

## 3. Results

A total of 253 patients were included in the study. The median age was 14 years (range 3–18), and 133 patients (52.6%) were female. Persistent subclinical inflammation was observed in 31 patients (12.3%). Genetic analysis revealed M694V homozygous mutation in 71 patients (28.1%). The median disease duration was 37 months (IQR 28–41) and median daily colchicine dose was 1 mg (IQR 1–1). The median hemoglobin level was 12.9 g/dL (IQR 12.1–13.6), median MCV was 82 fl (IQR 78–86), median neutrophil count was 3860/µL (IQR 3060–5050), and median lymphocyte count was 2550/µL (IQR 2040–3090). The median NLR was 1.57 (IQR 1.15–2.19). Regarding inflammatory parameters, the median CRP was 2 mg/dL (IQR 0.9–3.9), median ESR was 2 mm/hr (IQR 2–6), and median SAA was 1.02 mg/L (IQR 0.48–3.77). The median ferritin level was 25 ng/mL (IQR 15–45), and iron deficiency anemia was detected in 46 patients (18.2%). In the group with persistent subclinical inflammation, daily colchicine dose, neutrophil count, NLR, CRP, ESR, SAA and ferritin levels were all significantly higher (*p* < 0.001, *p* = 0.008, *p* = 0.018, *p* < 0.001, *p* < 0.001, *p* < 0.001, and *p* = 0.012, respectively). The median MCV was significantly lower in the persistent subclinical inflammation group (*p* = 0.002) **(Table 1)**.

Vitamin levels were as follows: median vitamin D 17.3 ng/mL (IQR 10.6–27.1), vitamin B12 288 pg/mL (IQR 214–367), and folate 6.4 ng/mL (IQR 4.8–7.6). Vitamin D levels were also categorized as deficient (<12), insufficient (12–20) or normal (>20), and then compared. When groups with and without subclinical inflammation were compared, no significant differences were found in vitamin levels (*p* = 0.658, *p* = 724, *p* = 0.449 and *p* = 0.113, respectively) **(Table 2)**.

## 4. Discussion

FMF is an autosomal recessive autoinflammatory disorder characterized by recurrent attacks, which may lead to severe complications such as amyloidosis in untreated patients. In some FMF cases, acute-phase reactants that markedly increase during attacks remain above normal even during attack-free periods, a condition defined as persistent subclinical inflammation. Previous studies have reported the prevalence of persistent systemic inflammation in FMF to range between 10 and 20% [2,3]. In our cohort, persistent subclinical inflammation was observed in 12.3% of patients, which is consistent with the existing literature.

Micronutrients have been shown to exert immunomodulatory effects in several immune-mediated diseases. Turhan T et al. [16] demonstrated a potential association between vitamin D receptor polymorphisms and FMF. In another study, serum vitamin B12 levels were found to be significantly lower in patients with autoimmune thyroid disease, with lower vitamin B12 levels being associated with higher anti-TPO titers [17]. In contrast, a study comparing patients with juvenile idiopathic arthritis and FMF to healthy controls reported lower vitamin D levels in the patient group, although no differences were observed between attack and remission periods [18]. Similarly, in another investigation assessing the relationship between micronutrient levels and FMF attack frequency, no significant correlation was found [12]. In line with these findings, our study also did not demonstrate significant differences in vitamin levels between patients with and without subclinical inflammation. Taken together, current evidence and our results suggest that while vitamin D deficiency appears to be more prevalent among FMF patients and may potentially contribute to disease pathogenesis, its association with disease activity is likely weak.

The lack of an observed association between micronutrient levels and persistent subclinical inflammation in our cohort may be explained by low-grade inflammation in FMF being primarily driven by genetically determined dysregulation of the innate immune system, particularly related to MEFV mutations and constitutive pyrin inflammasome activation [4]. This genetically driven inflammatory background may outweigh the relatively modest immunomodulatory effects of micronutrients, especially during attack-free periods.

In our cohort, micronutrient levels did not differ significantly between patients with and without persistent subclinical inflammation. Nevertheless, the relatively small number of cases with subclinical inflammation, together with the lack of adjustment for all potential confounding factors affecting vitamin levels, may have limited our capacity to detect a possible association between micronutrient status and subclinical inflammation.

The main limitations of our study are its retrospective design and single-center setting, which may restrict the generalizability of the findings. The lack of adjustment for seasonal variation in micronutrient levels due to the retrospective design of our study, as well as the inability to include body mass index data in the analysis because of missing values, were additional limitations of our study. It should also be considered that colchicine use may impair vitamin B12 absorption, which could limit the interpretation of the association between vitamin B12 levels and subclinical inflammation. Furthermore, considering the limited number of patients with subclinical inflammation and the relatively small differences observed in vitamin levels between groups, the statistical power of our study may have been insufficient to detect these differences. Prospective studies investigating the relationship between vitamin levels and subclinical inflammation in FMF are warranted to provide further insights into this issue.

## 5. Conclusions

Although the immunomodulatory effects of vitamin D, vitamin B12, and folate levels have been discussed in the literature, our study did not demonstrate an association between vitamin levels and subclinical inflammation in FMF patients with evidence of subclinical inflammation. Prospective studies on this topic may provide further contributions to the literature.

## Figures and Tables

**Table 1:** Demographic and laboratory parameters of the study group.

Variables	All Patients (N = 253)	Without Subclinical Inflammation (N = 222)	With Subclinical Inflammation (N = 31)	*p*-Value
**Age (Years, Median, Range)**	14 (3–18)	14 (3–18)	13 (4–18)	*0.142 ^a^*
**Sex (N, %)**				
Female	133 (52.6%)	119 (53.6%)	14 (45.2%)	*0.378 ^b^*
Male	120 (47.4%)	103 (46.4%)	17 (54.8%)	
**Genetic Mutations (N, %)**				
M694V homozygous	71 (28.1%)	58 (26.1%)	13 (41.9%)	*0.300 ^b^*
M694V heterozygous	59 (23.3%)	51 (23%)	8 (25.9%)	
Compound heterozygous other than M694V	23 (9.1%)	22 (9.9%)	1 (3.2%)	
Heterozygous other than M694V	48 (19%)	44 (19.8%)	4 (12.9%)	
Wild-type	52 (20.5%)	47 (21.2%)	5 (16.1%)	
**Disease Duration (Months, Median, IQR)**	37 (28–41)	36 (26–41)	38 (35–40)	*0.301 ^a^*
**Daily Colchicine Dose (mg, Median, IQR)**	1 (1–1)	1 (1–1)	1.5 (1.5–2)	***<0.001 ^a^***
**Hemoglobin (g/dL, Median, IQR)**	12.9 (12.1–13.6)	12.9 (12.1–13.8)	12.6 (11.5–13.2)	*0.142 ^a^*
**MCV (fL, Median, IQR)**	82 (78–86)	82 (79–86)	77 (75–82)	***0.002 ^a^***
**Neutrophil (/µL, Median, IQR)**	3860 (3060–5050)	3770 (2950–4908)	4400 (3780–5585)	***0.008 ^a^***
**Lymphocyte (/µL, Median, IQR)**	2550 (2040–3090)	2555 (2040–3088)	2330 (2020–3010)	*0.524 ^a^*
**NLR (Median, IQR)**	1.57 (1.15–2.19)	1.51 (1.12–2.17)	2.07 (1.33–2.64)	***0.018 ^a^***
**CRP (mg/dL, Median, IQR)**	2 (0.9–3.9)	1.7 (0.9–3.2)	8 (6–13.5)	***<0.001 ^a^***
**ESR (mm/h** **, Median, IQR)**	2 (2–6)	2 (2–6)	7 (2–21.5)	***<0.001 ^a^***
**SAA (mg/L, Median, IQR)**	1.02 (0.48–3.77)	0.98 (0.46–3.27)	7 (1.08–19.7)	***<0.001 ^a^***
**Ferritin (ng/mL, Median, IQR)**	25 (15–45)	23 (14–42)	36 (24–73)	***0.012 ^a^***
**Iron Deficiency Anemia (N, %)**				
Absent	207 (81.8%)	183 (82.4%)	24 (77.4%)	*0.498 ^b^*
Present	46 (18.2%)	39 (17.6%)	7 (22.6%)	

^a^ Mann–Whitney U-test, ^b^ Chi-square test. CRP: C-reactive protein, ESR: erythrocyte sedimentation rate, IQR: interquartile range, MCV: mean corpuscular volume, NLR: neutrophil–lymphocyte ratio, SAA: serum amyloid A. All *p*-values less than 0.05 are in bold.

**Table 2:** Vitamin levels according to subclinical inflammation status.

Variables	All Patients (N = 253)	Without Subclinical Inflammation (N = 222)	With Subclinical Inflammation (N = 31)	*p*-Value
**Vitamin D (ng/mL, Median, IQR)**	17.3 (10.6–27.1)	16.8 (10.6–27)	19.3 (12.5–27.3)	*0.658 ^a^*
**Vitamin D status**				
Deficiency	114 (45%)	102 (46%)	12 (38.7%)	*0.724 ^b^*
Insufficiency	99 (39.1%)	85 (38.3%)	14 (45.2%)	
Normal	40 (15.9%)	35 (15.7%)	5 (16.1%)	
**Vitamin B12 (pg/mL, Median, IQR)**	288 (214–367)	291 (215–366)	237 (210–364)	*0.449 ^a^*
**Folate (ng/mL, Median, IQR)**	6.4 (4.8–7.6)	6.4 (4.8–7.5)	6.6 (4.7–8.4)	*0.113 ^a^*

^a^ Mann–Whitney U-test, ^b^ Chi-square test. IQR: interquartile range.

## Data Availability

The datasets used and/or analyzed during the current study are available from the corresponding author on reasonable request.

## Abbreviations

The following abbreviations are used in this manuscript:

CRP	C-reactive protein
ESR	Erythrocyte sedimentation rate
FMF	Familial Mediterranean fever
MCV	Mean corpuscular volume
MEFV	Mediterranean fever
NLR	Neutrophil-to-lymphocyte ratio
SAA	Serum amyloid A
